# Social Determinants of Infectious Diseases in South Asia

**DOI:** 10.1155/2014/135243

**Published:** 2014-10-30

**Authors:** Ghose Bishwajit, Seydou Ide, Sharmistha Ghosh

**Affiliations:** ^1^School of Social Medicine and Health Management, Tongji Medical College, Wuhan, Hubei 430030, China; ^2^Faculty of Health Sciences, University of Ottawa, Ottawa, ON, Canada K1N 6N5; ^3^Department of Public Administration, Huazhong University of Science and Technology, Wuhan, Hubei 430074, China

## Abstract

South Asian countries have developed infectious disease control programs such as routine immunization, vaccination, and the provision of essential drugs which are operating nationwide in cooperation with many local and foreign NGOs. Most South Asian countries have a relatively low prevalence of HIV/AIDS until now, but issues like poverty, food insecurity, illiteracy, poor sanitation, and social stigma around AIDS are widespread and are creating formidable challenges to prevention of further spread of this epidemic. Besides that, resurgence of tuberculosis along with the emergence of the drug resistant (MDR-TB and XDRTB) strains and the coepidemic of TB and HIV are posing ever-growing threats to the underdeveloped healthcare infrastructure. The countries are undergoing an epidemiological transition where the disease burden is gradually shifting to noncommunicable diseases, but the infectious diseases still account for almost half of the total disease burden. Despite this huge burden of infectious diseases in South Asia, which is second only to Africa, there is yet any study on the social determinants of infectious diseases in a local context. This paper examines various issues surrounding the social determinants of infectious diseases in South Asian countries with a special reference to HIV and tuberculosis. And, by doing so, it attempts to provide a framework for formulating more efficient prevention and intervention strategies for the future.

## 1. Introduction

In the domain of public health, the term “social determinants” indicates the set of factors that contribute to the social patterning of health, disease, and illness which are referred to collectively as social determinants of health (SDOH). The Commission on Social Determinants of Health (CSDH) was established by WHO in March 2005 to support countries and global health partners to help address the social factors leading to illness and health inequality [1, 2]. The commission aimed to draw attention of the research sector, governments, and the academia to the social determinants of health and in creating better social conditions for health, particularly among the most vulnerable people [3]. South Asia, which is also known as the Indian subcontinent, is home to over one-fifth of the global population. Studies have shown that a major share of health problems is attributable to the integrated and overlapping socioeconomic factors [4–7]. The major social determinants that make countries vulnerable to infectious disease epidemics include poverty, illiteracy, gender inequality, and rapid urbanization. All of these factors are pervasive across South Asia and remained largely unaddressed to date. Even though chronic noncommunicable diseases (NCDs) are rapidly emerging in this region, infectious diseases still contribute to a significant portion of all disease burden [8]. Studies have suggested that TB patients were concentrated in areas with high population density and poor environmental and sanitation conditions [9, 10].

The infectious diseases of poverty continue to appear as major obstacles to attain the health related Millennium Development Goals (4, 5, and 6) [11] in South Asia. Though the healthcare system of South Asian countries has registered remarkable progress during past two decades, the benefits remain very unevenly shared. Infant survival rate in South Asia is still the second lowest in the world and the root causes are mostly nonmedical such as poverty, food insecurity, and other associated problems such as unhygienic living conditions and lack of pure drinking water [12]. Worldwide, an estimated 98 percent of children who die of pneumonia live in the developing countries and approximately 700,000 under five years die of pneumonia in South Asia [13].

Accumulating evidence suggests that not only is treating HIV and AIDS an action against the disease, but it is more about addressing the social and economic roots which are thwarting the prevention and intervention efforts. Studies have found that incidence rates are clearly higher in areas with average and lower socioeconomic levels and concluded that TB-HIV coinfection is a disease of social complexity, and the methods of elimination are limited not merely to health, but also on improving housing, transportation, and nutrition [14]. Though the rates of HIV and AIDS are still low compared to other developing regions, South Asia remains a high risk zone owing to inadequate concern regarding the social determinants. HIV has contributed to the rapid rise in the incidence and prevalence of tuberculosis, and TB/HIV coinfection has been found to reduce the effectiveness of DOTS (directly observed treatment, short course) programs in South Asia [15–17]. HIV significantly reduces the immune response to TB and increases vulnerability to TB infection. Thus the coexistence of TB/HIV leaves individuals at greater risk than any of the two diseases alone [18].

## 2. Social Context of Major Infectious Diseases in South Asia

**Figure 1:**
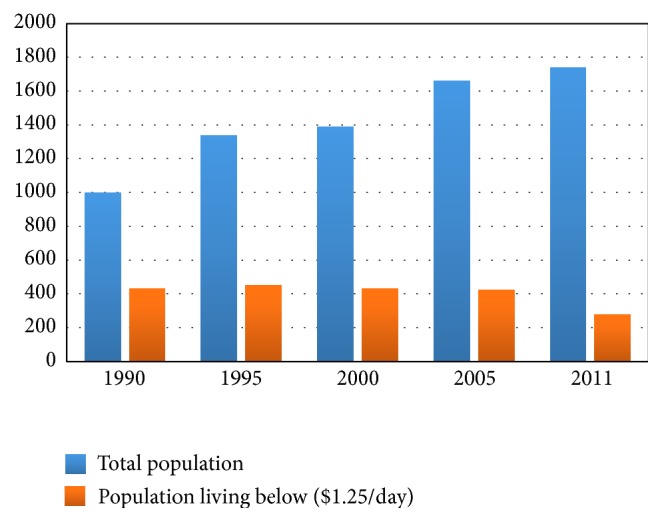
South Asia poverty dynamics. Figure 1 shows the trend of total population and population living below poverty line in South Asia. Though the incidence of poverty is decreasing slowly since 1995, it still remains noticeably high. Source: World Bank poverty database and Global Poverty Statistics.

In developed regions like Europe, the focus of majority of the researches is on socioeconomic determinants of chronic diseases such as diabetes, cancer, and cardiovascular disease since infectious diseases constitute a small fraction of the total disease burden (7%) [19]. In Africa, on the other hand, communicable diseases account for 63 percent of all deaths. Unfortunately, in countries where infectious diseases are rife such as in South Asia or Africa, there are no notable scientific studies on the socioeconomic determinants of infectious diseases. As economic unification and free trade are gaining momentum in Asia and Africa, it is also improving the overall living standard across the countries. However, growing evidence suggests that certain aspects of industrialization and cross-border trade are undermining the adverse impacts of climate change on public health, particularly in the poorest countries [20, 21]. Globalization and free trade have spurred economic growth in South Asian countries with substantial improvement in human development indices. Yet, despite a period of marked economic growth averaging 6 percent a year [20] over the past two decades, it remains world's second poorest region with around 400 million people living on less than $1.25 a day (Figure 1). In South Asia, a great majority of the infectious diseases can be attributed to the direct consequences of poverty such as poor nutritional status, overcrowded housing conditions, lack of access to healthcare, poor hygiene, and sanitation. According to WHO, diseases associated with poverty account for 45 percent of all diseases in the poorest countries and tuberculosis, malaria, and HIV/AIDS together are responsible for nearly 18 percent of the total disease burden [22]. Socioeconomic and environmental factors, such as poverty, polluted air, and water, are identified as very important risk factors for transmission of TB infection [23].* TB patients are shown to suffer impoverishment due to loss of income and consequently depending on selling household properties* [24]. In South Asia, the average TB patient loses around three to four months of work time and up to 30 percent of yearly household earnings [22].  Table 1 illustrates that the likelihood of dying from causes related to poor socioeconomic status such as scarcity of safe drinking water, poor sanitation, and unsafe sex is remarkably high in LMIC countries. Nearly one billion people in South Asia live without access to adequate sanitation and diarrhoea continues to be a leading cause of child deaths [25]. There is a strong relationship between poverty, squalid living environment, and the number and severity of diarrheal episodes, especially for children aged under 5 years. It is also estimated that access to safe drinking water and improved sanitation in Bangladesh could reduce diarrheal diseases by nearly 90 percent [22]. In the rapidly growing urban areas where population density is too high, the synergy between people's socioeconomic condition and nutritional status significantly increases their vulnerability to diseases such as tuberculosis and diarrhea [26].  Table 2 shows that number of deaths due to infectious diseases is much higher in the LMICs than in the high-income countries. Bangladesh is a highly flood-prone country and flooding has been shown to increase the prevalence of water-borne diseases during monsoon every year to which children are particularly susceptible. Diarrhea is responsible for one-third of the total child death burden in Bangladesh claiming around 230,000 lives in rural areas annually [27]. Malaria was nearly eradicated from India in the early 1960s but the disease has reemerged as a major public health problem and a great majority of people are now living in malaria-prone areas [28]. India was estimated to be the largest contributor of malaria to South and South East in 2009 and the estimated number of malaria cases in South Asian region was 90–167 million and number of estimated deaths was 125,000 per year [5].

## 3. TB Epidemic in South Asia

**Figure 2:**
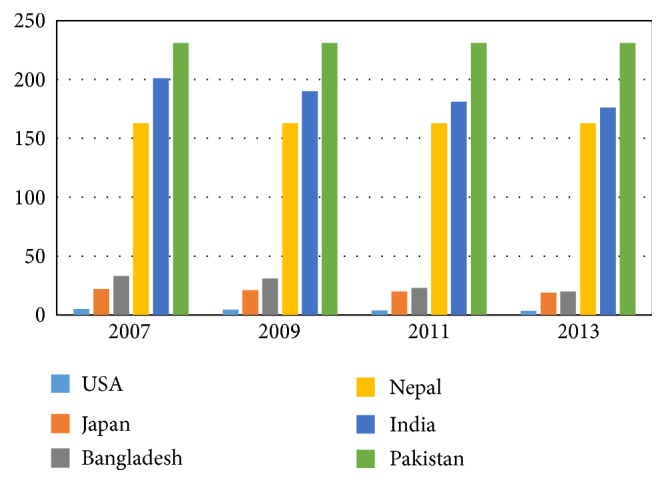
Incidence of TB per 1000 population.  Figure 2 illustrates that incidence of TB is remarkably lower in the developed countries like USA and Japan than in the third world countries like in South Asia. India and Bangladesh have one of the highest incidence rates of TB in the world. Source: World Health Statistics and Global Disease Burden.

Tuberculosis was once considered to be under control but has bounced back in full force as a leading infectious disease in many South Asian countries. TB is claimed to be one of the major diseases of poverty affecting the lives and livelihoods of most vulnerable population (above 90 percent of the global tuberculosis cases and deaths occur in the developing world) [29–32]. Prevalence ofTB is also shown to be greatly influenced by inequity in income distribution [33].  Figure 2 shows that TB is highly prevalent among all South Asian countries.The impact of poverty on higher rates of TB particularly in a low income country like India has been well depicted in previous studies (Table 3). The coexistence of TB and HIV along with the increasing rate of the MDR and XDR type tuberculosis is aggravating the situation and posing overwhelming challenges to national TB control programs (NTP) [34]. South Asian countries are struggling to control tuberculosis through the implementation of WHO's DOTS (directly observed therapy short course) strategy [12]. Though many other strategies were introduced besides the DOTS, poor public health infrastructure, staff shortages, inadequate funding, and lack of awareness about the strategy among private practitioners remain the main constraints to the successful implementation of these strategies [12, 35].

Burden of TB in Bangladesh is one of the highest in the world with an estimated incidence of 353,000 in 2007 which is the sixth highest in global ranking [36] and ninth among 25 high priority MDR and XDR countries [37]. The National Tuberculosis Control Programme of Bangladesh first adopted the DOTS strategy in 1993. Since then program rapidly expanded to almost all areas of the country reaching 100 percent coverage in 2006 [38]. Though India had a National Tuberculosis Programme in place since 1960, tuberculosis remains a major public health problem accounting for around one-fifth of all tuberculosis cases reported globally. India is also facing converging dual epidemics of TB and HIV. The National AIDS Control Organization has taken a decision to routinely offer HIV testing to all diagnosed TB patients in the high-prevalence states [39]. Nepal lies between two high TB burden countries, India and China, which together account for one-third of the world's TB cases [40]. In 1995, the National Tuberculosis Control Programme of Nepal adopted the DOTS strategy and since then the private health care providers are encouraging tuberculosis suspect patients to seek care from this program [41]. The DOTS centers in Nepal provide free of charge treatment which includes two months intensive treatment under direct observation and six months treatment in continuation phase. Tuberculosis is also a huge public health issue in Pakistan and ranks fifth among high tuberculosis burden countries in the world. Though the DOTS strategy was implemented in Pakistan in 2001, the detection and treatment programs in the country suffer many constraints owing to complex emergency situations including humanitarian crises and conflicts [8]. Prevalence of MDR and XDR strains is also high in Pakistan. It is true that improved diagnosis and treatment through the DOTS strategy have saved millions of lives. However, their impact on TB incidence has been dissatisfactory and the prevalence remains overwhelmingly high in most South Asian countries.

## 4. HIV/AIDS Trajectory in South Asia

**Figure 3:**
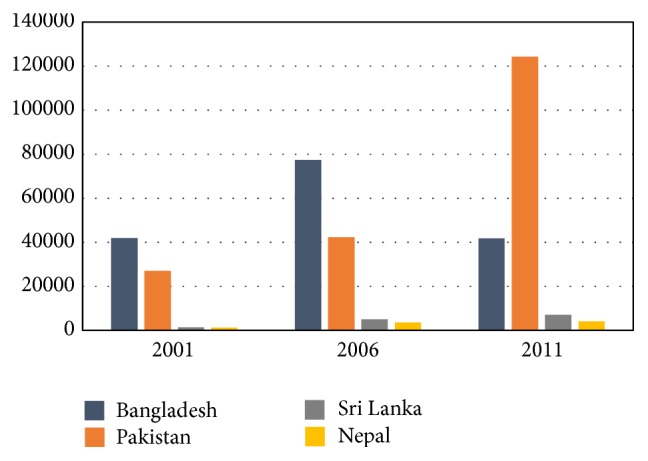
Prevalence of HIV among adults aged 15–49.  Figure 3 shows that Pakistan had about 130,000 cases of HIV in 2011 which is the highest in the history of the country. Nepal and Sri Lanka have relatively lower incidence of HIV comparing to Pakistan and Bangladesh. Source: adapted from World Health Statistics.

The first World AIDS day was observed on first of December in 1988 by taking global commitments and with the red ribbon worn marking the battle against the global epidemic. Despite such comprehensive efforts, it has spread rapidly across the globe especially in the poor countries like in Africa, South East, and South Asia. According to WHO, around 500,000 adults and children in South Asia were newly infected with HIV in 2002. Still now, South Asian countries have relatively low HIV prevalence rates, but prevalence is growing rapidly among groups at high risk such as sex workers and their clients, men having sex with men (MSM), and injecting drug users and their partners. Today, around more than 6 million people in South Asia are living with HIV/AIDS and four out of every five of them live in India. The first AIDS case in India was detected in 1986 [42]. Today, India has highest HIV prevalence in South Asia followed by Pakistan and Nepal [43]. However in recent years the high prevalence states in India showed a declining trend in adult HIV prevalence and in 2011 the estimated annual new HIV infection was 0.116 million which is 57 percent lower compared to the figure in 2000. By the end of 2012, around 2.39 million Indians were reported to be living with HIV making it home to world's third largest HIV infected population [44].  Figure 3 illustrates the trend in HIV prevalence in four South Asian countries and reveals that Bangladesh has relatively low prevalence of AIDS in South Asia. Despite that, the country remains extremely vulnerable to HIV epidemic due chiefly to widespread poverty, overpopulation, gender and health inequality, social stigmatization, and high rates of commercial sex.

In Bangladesh the first case of HIV/AIDS was detected in 1989 [44]. UNAIDS estimates that about 12,000 Bangladeshis were living with HIV at the end of 2007 [43]. Currently there are around 380 NGOs and AIDS service organizations are currently involved in AIDS related programs in the country [45]. The first AIDS case in Nepal was reported in 1988 and, as of 2011, national estimates indicated that about 49,000 adults and children are affected with HIV. By the middle of 2008, more than 1750 cases of AIDS and over 11,000 cases of HIV infection were officially reported in Nepal [44].

The first AIDS case in Pakistan was reported in 1987 [46] and the number of reported cases of HIV/AIDS has been continuously increasing since then. HIV prevalence almost doubled in the period between 2005 and 2008 from 11 to 21 percent and today Pakistan has the second highest prevalence of HIV in South Asia. The National AIDS Control Program (NCP) is one of the pioneering institutions in Pakistan which provides free treatment to HIV/Aids patients through 20 AIDS treatment centers in the country [46]. Sri Lanka's HIV epidemic is considered low-level with an estimated 4632 people living with HIV in 2012 and in total 283 AIDS-related deaths are reported to date since the detection of first case in 1987 [24]. Sri Lanka has the most efficient blood screening process in South Asia which has contributed greatly to maintain a low prevalence rate of HIV. According to local specialists there have been no incidents of HIV infection via blood transfusion in the country since 2000, while in other South Asian nations reused syringe and unsafe blood transfusion remain major routes of infection.

## 5. NTDs

The NTDs represent a group of 17 parasitic, bacterial, and viral infections among impoverished and disadvantaged populations in developing countries. Globally, NTDs affect around a billion and kill around 9 million people each year. These diseases occur primarily in rural and poor urban areas of South Asia, sub-Saharan Africa, and Latin America [54]. NTDs are found to be nearly nonexistent among the industrialized countries and the wealthy societies in the developing countries [55]. This disproportionate burden of NTDs among the poor is attributed to various sociodemographic and economic determinants. The social pathways of becoming infected with NTDs include socially determined factors including illiteracy, malnutrition, poor living conditions, and unemployment [56]. According to WHO, the analysis of social determinants of the NTDs is extraordinarily complex due to their heterogeneous nature and diverse social determinant profile and thereby proposes six broad strategic frameworks (Table 4) to tackle them. The burden of NTDs is huge in South Asia compared to other developing regions of the world and is rising constantly due to widespread prevalence of risk factors and lack of effective treatment opportunities. Most common NTDs in South Asia include ascariasis, trichuriasis, hookworm infection, lymphatic filariasis, and leishmaniasis.

Apart from causing lives, NTDs have serious impacts on household income and productivity. A study of lymphatic filariasis (LF) in India showed that every year $842 million are lost due to treatment costs and reduced working time which is equivalent to $2 per person resident in endemic areas [57]. Research conducted in Sri Lanka showed that women with LF can lose their jobs and be abandoned by their families [58]. While poverty seems to be the most prominent of social determinants of NTDs, health illiteracy and superstitions are also very strongly associated. People in rural areas often believe that diseases are the will of nature and cannot be cured by human efforts. Besides that, most villagers depend on traditional treatment methods which are hardly effective against rare types of infectious diseases. Persistent poverty and social inequality have been shown to be responsible for reemergence of NTDs [59].

Almost all South Asian countries face a heavy burden of visceral leishmaniasis which is also known as kala-azar. This disease has actually reemerged from a near eradication state to account for nearly 80 percent of the cases occurring globally. Bihar alone accounts for half of the world's annual new cases. The widespread coexistence of kala-azar and other infectious diseases poses additional complications for accurate diagnosis and proper treatment. In Bangladesh, the major types of NTDs are lymphatic filariasis, kala-azar, and soil transmitted helminthiasis. Poor living conditions, lack of access to safe drinking water, poor sanitation, and sewerage are responsible for transmission of vectors of NTDs. Living in proximity to a kala-azar case is the strongest risk factor for disease in Bangladesh. In India, leptospirosis happens to be prevalent among those who work mostly in polluted conditions such as farmers [60]. South Asia accounts for around one-quarter of the world's soil-transmitted helminthiases cases, with the largest number of cases in India, followed by Bangladesh [61]. Leptospirosis, a worldwide zoonosis associated with sinister complications and fatalities, has been recognized in India since 1931 [61]. Poverty is a key determinant of leishmaniasis [62] and it is especially rampant in southern, central, eastern, and western India, where heavy monsoon, animal rearing practices, and agrarian way of life predispose to this infection [63]. NTDs are diseases of socially excluded populations that promote poverty by relatively depriving individuals from basic capabilities [55]. These diseases pose enormous challenges to development through impacting livelihoods, physical and socioeconomic wellbeing. Infectious agents deplete body's nutrient pool by affecting nutrient metabolism which debilitates the immune system. If these diseases remain unnoticed and untreated, the poverty alleviation and human development goals are unlikely to be achieved. Lack of access to healthcare, health illiteracy, social stigma, and poor diet also contribute to increase vulnerability to NTDs and thwart prevention efforts. NTDs are identified to be a major impediment to achieving Millennium Development Goals (MDGs) and comprehensive programs to eliminate some of the highly prevalent NTDs are under way in South Asia [61]. NTDs are largely preventable diseases provided their social roots are adequately understood and addressed. Addressing these social determinants of NTDs in synergy with the existing tools to combat NTDs can prove highly successful.

## 6. Understanding the Social Determinants of Infectious Diseases in South Asia

Accumulating evidence suggests that the social context in which individuals live and work has great influence on health and wellness [7, 64]. However there remains a huge lack in our comprehension concerning those contexts and the extent to which they influence the diseases and their underlying causes. Since societies differ significantly in terms of economic prosperity, cultural and geographical setting, type and quantity of food grown, and taste and diet pattern and since various diseases thrive in various environments, it is therefore critical to consider the contextual factors specific to the particular country or geographic region while designing strategies for intervention of diseases. Evidences suggest that poor countries require not only investment in strengthening tuberculosis control programs, diagnostics, and treatment but also action on the social determinants of tuberculosis [23]. Therefore, besides innovating better intervention technologies, there is also a crucial need to address the societal factors that determine the course of these diseases and the risk factors [65].

**Figure 4:**
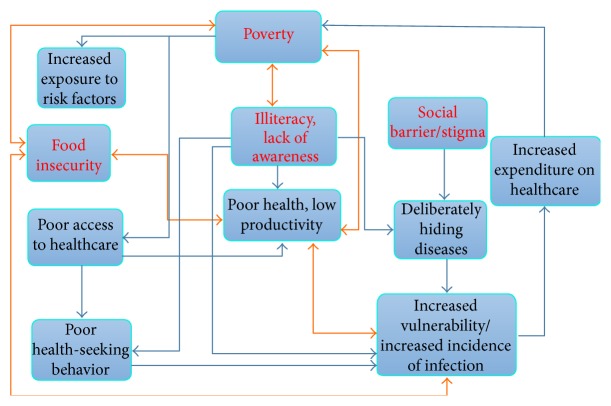
The nexus between the social determinants and the infectious disease. Four main social determinants of the infectious diseases are shown in the red letters.

Poverty and food insecurity are by far two most widely studied topics in relation to both communicable and noncommunicable diseases in the developed countries. In South Asia, the causes of most infectious diseases (especially TB, diarrhea, and malaria) are closely associated with poverty, poor sanitation, and food insecurity and there exists a destructive link between the social factors and the occurrence of these diseases.  Figure 4 gives a basic outline of the cyclical relationship between the social determinants and the infectious diseases. Studies have shown that household food insecurity heightens the vulnerability to HIV transmission behaviors and susceptibility to HIV infection [66, 67]. Table 3 lists some of the major studies that demonstrate the impact of poverty on the spread of TB and helps to understand why the incidence is much higher among the poorest. Considering the rising trends, tackling the infectious diseases in South Asia remains a big challenge for the governments and the private sectors. Addressing the social determinants is certainly going to be a colossal task which will require high level and interdisciplinary policy making and strong commitment by the stakeholders at all levels to implement and assess the outcome of the policies. The governments in South Asian countries are very unlikely to succeed in improving the situation single-handedly and working in conjunction with the NGOs can substantially reduce the burden on healthcare systems and enhance the efficacy of such programs as well since the NGOs have shown better performance in reaching the most disadvantaged communities. The microcredit program in Bangladesh for TB treatment has shown promising outcomes in recent years and many other countries are now working on increasing the provision of microcredits among the most vulnerable population. In 2006, 10.3 percent of total TB patients received microcredit support from BRAC (Bangladesh Rural Advancement Committee), and report says that the loan has decreased the prevalence of TB in the target population [68]. Apart from resulting in loss of lives, TB and AIDS cause substantial economic losses especially among adults of working age and adult ill health is a major cause of impoverishment of many families [15]. People living with HIV infection face not only illness but also poor productivity and reduced income and end up making difficult choices among essential but competing expenses, such as food versus health care and education versus housing [69]. According to a World Bank report, average annual expenditure for treatment of a person living with AIDS is higher than educating ten primary school students in India. Health literacy is another very important factor as poor and illiterate people tend not to respond equally well to health programs compared with their literate counterparts [70]. Illiterate families are also very likely to neglect the members infected with HIV which increases the risk of further spreading. Since HIV is also a social disease, part of the treatment process also lies in the society which involves having a good social status, psychosocial support from family and society, and equal access to health services. Many other aspects of these diseases are entirely social with which healthcare system has nothing to do. There remains a coresponsibility for policy makers, the advanced academia, and the civil society as well to reshape the social and ideological paradigm which will not only support people's right to health and well-being, but also provide the conditions which are crucial to exert their right to health and live a healthy life. Thus it can be assumed that the future prevalence of TB and HIV in South Asia will be determined by the extent to which they will be able to improve the social factors such as people's living status, literacy, availability of food and clean water, and the performance of the healthcare system in incorporating the strategies to address the socioeconomic determinants of health into the mainstream healthcare [1].

## 7. Conclusion

Diseases do not discriminate people, but people do, which causes a disproportionate disease burden on the discriminated. Mere indexing of the “diseases of affluence” and the “diseases of poverty” is not going to reduce the burden of the infectious diseases that afflict poor countries unless the underlying social, cultural, and attitudinal constraints are addressed with due priority. South Asian countries need to formulate strategic social and health policies to promote both quality and equity in healthcare service and for which emphasis should be assigned on the social factors, especially on ensuring social stability, enhancing social inclusion and community involvement in order to broaden public understanding of social issues in mitigating the health problems. Reducing discrimination and stigmatization with regard to the infectious diseases and focusing on the most vulnerable groups especially women and children are of crucial importance and can be instrumental in minimizing and preventing further spread of the life threatening but preventable diseases like TB and AIDS. This paper concludes that addressing the social determinants is absolutely essential for sustainable intervention and prevention of further spread of the infectious diseases especially TB and HIV which are currently and likely to remain two major public health concerns in the region.

## 8. Limitations of the Study

This study has few limitations which are due principally to unavailability of sufficient and reliable data. Firstly it includes only India, Pakistan, Nepal, Bangladesh, and Sri Lanka partly because these five countries constitute about 87 percent of the total population in South Asia and show somewhat similar health and disease pattern. Secondly, considering the rate of incidence and the future implications on society and the economy, this paper focuses mainly on TB and HIV, but there are many other infectious diseases on the list which are also regarded very serious such as viral hepatitis, malaria, and pneumonia.

## Figures and Tables

**Table 1:** Comparison of total number of deaths attributable to risk factors between high-income countries and low-and-middle income countries (LMICs) in 2001. It is clear that, in LMICs, people are more at risk of dying from the causes which are influenced by lower socioeconomic status and lack of awareness about these diseases.

Risk factor	LMICs		High-income countries	
	Total number of deaths	Percent	Total number of deaths	Percent
Childhood underweight	3,630	7.5	0	0
Poor sanitation and unclean water	1,563	3.2	4	<0.1
Smoking	3,340	6.9	1,462	18.5
Unsafe sex	2,819	5.8	32	0.4
Contaminated injections	407	0.8	4	<0.1

Source: Global Disease Burden, 2001.

**Table 2:** Total number and percentages of deaths in low-and-middle income countries (LMICs) caused by five major infectious diseases.

Causes of deaths	LMICs		High-income countries	
	Total number of deaths	Percent	Total number of deaths	Percent
Tuberculosis	1,590	3.3	16	0.2
HIV/AIDS	2,552	5.3	22	0.3
Diarrhea	1,777	3.7	6	<0.1
Measles	762	1.6	1	<0.1
Malaria	1,207	2.5	0	0

Source: Global Disease Burden, 2001.

**Table 3:** Selection of studies demonstrating the influence of poverty on tuberculosis.

Study title	Reference	Result
Tuberculosis and poverty	Spence et al. [47]	Tuberculosis remains strongly associated with poverty

Tuberculosis and poverty: why are the poor at greater risk in India?	Oxlade et al. [48]	TB control strategies should be targeted to the poorest populations that are most at risk and should address the most important determinants of disease

Cash transfer and microfinance interventions can positively impact TB risk factors	Boccia et al. [49]	Cash transfer and microfinance interventions can positively impact TB risk factors

The Innovative Socioeconomic Interventions Against Tuberculosis (ISIAT) project: an operational assessment	Rocha et al. [50]	The Innovative Socioeconomic Interventions Against Tuberculosis (ISIAT) project: an operational assessment

The economic burden of tuberculosis care for patients and households in Africa: a systematic review	Ukwaja et al. [51]	The average patient's/household's prediagnostic costs for TB care were catastrophic

Addressing poverty through disease control programmes: examples from tuberculosis control in India	Kamineni et al. [52]	Further in-depth analysis as well as systems/policy/operations research exploring pro-poor initiatives, in particular examining service delivery synergies between existing poverty alleviation schemes and TB control programme, is essential

The association between household poverty rates and tuberculosis case notification rates in Cambodia, 2010	Wong et al. [53]	There is a positive association between household poverty rates and sputum-positive TB

**Table 4:** Strategies proposed by WHO to address the social determinants of NTDs.

Number	Social determinants	Strategies
1	Potable water and sanitation	Identifying the links between social determinants of access to water and sanitation in regard to NTDs
2	Environmental factors	Showing the impact of environmental variables on NTDs
3	Health service for migrating populations	Making better policies to reduce the vulnerability of migrant labors, nomads, refugees, and disaster victims to NTDs
4	Sociocultural and gender inequality	Identifying sociocultural factors that lead to unequal access to NTDs treatment
5	Poverty	Poverty alleviation as a strategy to reduce the incidence of NTDs
6	Risk assessment and surveillance	Establishing cross-disciplinary risk assessment, surveillance systems, early warning, data accumulation, and long term planning

## Abbreviations

BRAC: Bangladesh Rural Advancement Committee
CSDH: Commission on Social Determinants of Health
DOTS: Directly observed treatment, short course
LIC: Low income countries
LMIC: Low-and-middle income countries
MDR: Multidrug resistant
NTDs: Neglected tropical diseases
NTP: National Tuberculosis Programme
SDOH: Social determinants of health
XDR: Extensively drug resistant.
